# Factors associated with viral RNA shedding and evaluation of potential viral infectivity at returning to school in influenza outpatients after treatment with baloxavir marboxil and neuraminidase inhibitors during 2013/2014–2019/2020 seasons in Japan: an observational study

**DOI:** 10.1186/s12879-023-08140-z

**Published:** 2023-03-29

**Authors:** Jiaming Li, Keita Wagatsuma, Yuyang Sun, Isamu Sato, Takashi Kawashima, Tadashi Saito, Yasushi Shimada, Yasuhiko Ono, Fujio Kakuya, Nobuo Nagata, Michiyoshi Minato, Naoki Kodo, Eitaro Suzuki, Akito Kitano, Toshihiro Tanaka, Satoshi Aoki, Irina Chon, Wint Wint Phyu, Hisami Watanabe, Reiko Saito

**Affiliations:** 1 Division of International Health (Public Health), Graduate School of Medical and Dental Sciences, Niigata University, Niigata, Niigata 951-8510 Japan; 2 Yoiko Pediatric Clinic, Niigata, Japan; 3 Kawashima Internal Medicine Clinic, Gunma, Japan; 4 Tako Central Hospital, Chiba, Japan; 5 Shimada Children’s Clinic, Kumamoto, Japan; 6 Ono Pediatric Clinic, Nagasaki, Japan; 7 Furano Kyokai Hospital, Hokkaido, Japan; 8 Hiraoka-Kouen Pediatric Clinic, Hokkaido, Japan; 9 Minato Pediatric Clinic, Tokyo, Japan; 10 Kodo Pediatric Clinic, Kyoto, Japan; 11 Suzuki Pediatric Clinic, Yamaguchi, Japan; 12 Kitano Pediatric Clinic, Kumamoto, Japan; 13 Shizuoka Welfare Hospital, Shizuoka, Japan; 14 Aoki Pediatric Clinic, Nara, Japan

**Keywords:** Influenza virus, Baloxavir marboxil, Neuraminidase inhibitor, Viral RNA shedding, Daily viral reduction

## Abstract

**Background:**

This study assessed the differences in daily virus reduction and the residual infectivity after the recommended home stay period in Japan in patients infected with influenza and treated with baloxavir (BA), laninamivir (LA), oseltamivir (OS), and zanamivir (ZA).

**Methods:**

We conducted an observational study on children and adults at 13 outpatient clinics in 11 prefectures in Japan during seven influenza seasons from 2013/2014 to 2019/2020. Virus samples were collected twice from influenza rapid test-positive patients at the first and second visit 4–5 days after the start of treatment. The viral RNA shedding was quantified using quantitative RT-PCR. Neuraminidase (NA) and polymerase acidic (PA) variant viruses that reduce susceptibility to NA inhibitors and BA, respectively, were screened using RT-PCR and genetic sequencing. Daily estimated viral reduction was evaluated using univariate and multivariate analyses for the factors such as age, treatment, vaccination status, or the emergence of PA or NA variants. The potential infectivity of the viral RNA shedding at the second visit samples was determined using the Receiver Operator Curve based on the positivity of virus isolation.

**Results:**

Among 518 patients, 465 (80.0%) and 116 (20.0%) were infected with influenza A (189 with BA, 58 with LA, 181 with OS, 37 with ZA) and influenza B (39 with BA, 10 with LA, 52 with OS, 15 with ZA). The emergence of 21 PA variants in influenza A was detected after BA treatment, but NA variants were not detected after NAIs treatment. Multiple linear regression analysis showed that the daily viral RNA shedding reduction in patients was slower in the two NAIs (OS and LA) than in BA, influenza B infection, aged 0–5 years, or the emergence of PA variants. The residual viral RNA shedding potentially infectious was detected in approximately 10–30% of the patients aged 6–18 years after five days of onset.

**Conclusions:**

Viral clearance differed by age, type of influenza, choice of treatment, and susceptibility to BA. Additionally, the recommended homestay period in Japan seemed insufficient, but reduced viral spread to some extent since most school-age patients became non-infectious after 5 days of onset.

**Supplementary Information:**

The online version contains supplementary material available at 10.1186/s12879-023-08140-z.

## Background

Influenza (the flu) is a respiratory infectious disease caused by influenza viruses that can cause seasonal epidemics annually. Seasonal epidemics are mainly caused by influenza A and B viruses. The World Health Organization (WHO) estimates that seasonal influenza results in 250,000 to 500,000 deaths annually [1], which is a severe burden on public health worldwide. Therefore, the effective treatment and control of the spread of influenza are crucial issues.

Neuraminidase inhibitors (NAIs) have been widely used to treat influenza in Japan [2]. In 2000, oseltamivir and zanamivir were approved for influenza treatment, while peramivir and lanimivir were approved in 2010. In 2018, baloxavir marboxil (baloxavir), a cap-dependent endonuclease inhibitor with a strong antiviral effect, was approved for influenza A and B [3], with reports of a significantly reduced viral titer after treatment [4, 5]. However, in clinical trials, the viral RNA shedding of baloxavir was compared only with oseltamivir [5–7] and not with other NAIs, such as lanimivir or zanamivir. Therefore, it is necessary to compare its viral RNA shedding with other NAIs.

Notably, it is reported that the emergence of antiviral-resistant viruses can delay viral clearance [8]. For NAIs, oseltamivir-treated patients developed resistance 0.4 to 4.0% of post-treatment isolates from adults and in 3.0 to 37.0% of isolates from children [9]. The viruses with NA gene substitution H275Y (NA/H275Y) are the most commonly resistant in A/H1N1pdm09, and the viral clearance was reported to be delayed [10]. Likewise, in baloxavir-treated patients, viruses with polymerase acidic (PA) gene substitutions of I38T/F/M were shed at a rate of 2.2% in phase II studies and 9.7% in phase III studies, and those viruses showed reduced susceptibility to baloxavir [5, 11]. In an open-label study limited to pediatric patients, viruses with PA gene substitutions of I38T/F/M shed at a rate higher than 23.4% [12], and the subsequent observational studies reported 3.8–41.0% emergence of PA variants (such as PA/E23G/K, I38F/M/K/S/T, or E119D) after treatment [6, 13–17]. Additionally, the rebound of viral RNA shedding was observed in the patients who developed these PA variant viruses after baloxavir treatment [12, 16]. Thus, the emergence of antiviral resistance is to be considered when evaluating the viral clearance in patients who received anti-influenza treatment.

Japanese schools have a mandated “stay at home” period when children are infected with influenza to avoid its spread. The School Health and Safety Act of Japan, enforced in 2012, stipulates that schoolchildren should stay at home for at least five days after the onset of illness, regardless of the timing of defervescence [18]. This 6 day-stay at home rule (including the day of onset) is applied not only to schoolchildren but also to students in high schools, colleges, universities and adults in many workplaces in Japan. However, few studies have evaluated the residual viral RNA shedding of patients after returning to school or workplace [19]. Therefore, evaluating the residual status of the influenza virus after five days of onset is necessary to determine the adequacy of the duration of school and workplace absence recommended in the School Health and Safety Act.

In the present study, we assessed the differences in daily viral reduction calculated from two-point samplings in influenza outpatients after receiving treatment with either cap-dependent endonuclease inhibitor, baloxavir, or three NAIs (laninamivir, oseltamivir, and zanamivir). We also examined whether the emergence of viruses with reduced susceptibility to baloxavir or NAIs could affect viral RNA shedding. Furthermore, we evaluated the potential viral infectivity in influenza A or B infected patients after 5 days of onset by viral RNA shedding in the second visit samples, using the potentially infectious cut-off values estimated from the virus isolation to determine whether the recommended absentee time in the School Health and Safety Act is sufficient.

## Methods

### Patient enrollment and treatment

Patients with influenza-like symptoms (such as fever, sore throat, cough, sneeze, or general fatigue) who visited 13 outpatient clinics in 11 prefectures of Japan (Hokkaido, Niigata, Gunma, Tokyo, Chiba, Shizuoka, Kyoto, Nara, Yamaguchi, Kumamoto, and Nagasaki) between 2013 to 2020 were enrolled in this study. First, patients were screened using influenza rapid diagnostic test (RDT) kits (QuickNavi-Flu + RSV™; Denka Co., Ltd, Tokyo, Japan). Written informed consent was obtained from patients with influenza or their guardians before enrolment. Then the clinicians collected the first sample, and prescribed one of the five drugs (baloxavir, laninamivir, oseltamivir, peramivir, or zanamivir) based on the advice of clinicians and/or the preference of patients or their guardians. However, due to the small number of patients treated with peramivir, those who received this medication were not included in the analysis. The dosage of the four drugs followed the standard prescription course recommended in Japan (Additional file 1: Table S1) [2].

### Collection of specimens and clinical data

Nasopharyngeal swabs or nasal discharges were collected from the patients in pairs at the first clinic visit before the start of treatment (pre-treatment) and at the second visit 4–5 days after the first visit (post-treatment). Clinicians recorded the patients’ age, sex, vaccination status, and illness onset dates during the first and second clinic visits. Samples were placed in viral transport media and frozen at − 20 °C at the study sites. Then, the samples were sent to Niigata University (Niigata, Japan) and stored at − 80 °C for further virologic examination.

### Quantitative real-time PCR for viral RNA shedding measurement

Viral RNA was directly extracted from the clinical samples collected during 2013/2014 and 2018/2019 season, using an Extragen II—DNA/RNA extraction kit (Tosoh Co., Ltd, Tokyo, Japan), and those collected in 2019/2020 season using QIAamp Viral RNA Mini Kit (Qiagen, Hilden, Germany) following the manufacturer’s instructions [14]. Viral RNA was then transcribed into complementary DNA (cDNA) using the Uni12 and Uni11 influenza A and B generic primers (Additional file 1: Methods) [14, 20].

Quantitative real-time PCR (RT-qPCR) targeting the M gene using TaqMan probes was carried out for the pre-and post-treatment clinical samples to detect viral RNA shedding of influenza A or B (Additional file 1: Methods). The detection limit was 0.447 log_10_ copies/µL (2.86 copies/µL) for influenza A and 0.462 log_10_ copies/µL (2.9 copies/µL) for influenza B [19].

### Viral clearance calculation

The daily viral reduction (viral clearance) in each patient was calculated from the viral RNA shedding in the paired first and second samples as follows:

$$Daily \,viral\, reduction=\frac{1}{t}\mathit{ln}\left(\frac{{\nu }_{0}}{{\nu }_{t}}\right)$$

where *t* is the number of days between the first and second visit, *v*_0_ and *v*_t_ stand for the viral RNA shedding at the first (pre-treatment) and second visits (post-treatment), respectively [21].

### Virus isolation to detect emergent NA/H275Y variant and to assess potential viral infectivity

Clinical samples (100 μL) were inoculated in confluent Madin-Darby canine kidney (MDCK) cells during the 2013/2014 and 2018/2019 seasons and SIAT-MDCK cells in 2019/2020 season propagated in 48-well plates to isolate the influenza viruses. Globally from 2018, isolating influenza A/H3N2 from MDCK cells became difficult; therefore, we changed the cells to MDCK-SIAT 1 that can proliferate A/H3N2 more easily [22, 23]. The 48-well plates were then incubated at 34 °C with 5% CO_2_ and observed daily for 5 days to detect the specific cytopathic effect (CPE) [24]. In addition, the viral isolates were used to detect NA/H275Y variant in A/H1N1pdm09 in the first and second visit samples. Although there is a possibility that viruses with low fitness, such as antiviral-resistant viruses, may be lost during the virus isolation, we assumed that the virus isolation is useful to see whether the measured viral RNA shedding contains active viral particles. Thus, the positivity or negativity of the viral isolation was used to assess potential viral infectivity in the concordant clinical samples with their viral RNA shedding [25].

### Screening of the NA/H275Y and PA/I38T variants by cycling probe RT-PCR assay

For the rapid detection of the NA/H275Y variant in A/H1N1pdm09, after RNA extraction, a cycling probe real-time PCR developed by our group was implemented on virus isolates for both pre- and post-treatment samples as previously reported [26]. For screening PA/I38T variants in both A/H1N1pdm09 and A/H3N2, a different set of cycling probe real-time PCR assay targeting at PA gene was implemented on the pre- and post-treatment clinical samples, as previously reported [14, 27].

### Genetic analysis to confirm NAIs or baloxavir-resistant variants

Genetic sequencing was conducted using the Sanger method to confirm the presence of amino acid substitutions in NA and PA genes that confer resistance to baloxavir or NAIs (Additional file 1: Methods) [14, 27, 28]. Genetic sequencing of NA gene was conducted on all isolates generated on MDCK or MDCK-SIAT 1 cells throughout the study period, and that of PA gene on all clinical samples collected between 2018/2019 and 2019/2020 seasons [14, 27, 28].

### Analysis of patient characteristics

Patient characteristics were analyzed as follows: age group (0–5 years, 6–18 years, ≥ 19 years), sex (male or female), treatment (baloxavir, laninamivir, oseltamivir, or zanamivir), influenza vaccination status (unvaccinated or vaccinated), drug resistance substitution (NA/H275Y or PA variants), viral RNA shedding at the first and second visits, interval time (from onset to first and second visits), all divided for analysis by influenza A or B. For the variables under investigation, the mean ± standard deviation (SD), median (interquartile range [IQR]), and/or frequency (%) were described. Student’s* t*-test, *χ*^2^ test, and Mann–Whitney *U* tests were used for the statistical analysis. All statistical analyses in this study were performed using EZR version 1.54 (Saitama Medical Center, Jichi Medical University, Japan) [29]. A two-sided *p*-value of less than 0.05 was considered statistically significant.

### Viral clearance between influenza A and B

The median values of daily viral reduction (i.e., viral clearance) were compared in the relevant categories of age groups, treatment groups, and influenza vaccination status using the Mann–Whitney *U* test to compare the difference between influenza A and B. Note that PA variants were detected only for influenza A, so the viral clearance was compared between those with or without emergent PA variants in influenza A, but not for influenza B.

### Univariate analysis of viral clearance by various factors

The median value of viral clearance was compared among age groups (0–5 years, 6–18 years, ≥ 19 years), treatment groups (baloxavir, laninamivir, oseltamivir, or zanamivir), vaccination status (unvaccinated or vaccinated), and the emergence of PA variants (yes or no), all divided by influenza A or B for univariate analysis. Mann–Whitney test was used to compare two groups, and the Kruskal–Wallis test was used to compare three or more groups. Bonferroni correction was applied as a post hoc test for each pair after the Kruskal–Wallis test.

### Multivariate analysis of viral clearance

The association between viral clearance and type of influenza (categorical variable), age group (categorical variable), treatment (categorical variable), vaccination status (categorical variable), the emergence of PA variants (categorical variable), and interval time from onset to first visit (continuous variable) were assessed using multiple linear regression analysis. We used the forced entry method for all examined variables (potential confounders) in the multivariate model. The maximum likelihood method was used for inference, and the estimates, including adjusted linear regression *β* coefficients, and standard error (SE), *t*-value, and *p*-value, were estimated. Goodness-of-fit was assessed by estimating the adjusted coefficient of determination (*R*^2^), *F-*value, and variance inflation factor (VIF).

### Assessment of residual viral RNA shedding at the time of returning to school

We analyzed patients' residual viral RNA shedding and potential viral infectivity after 5 days of onset. The onset date was regarded as day 0. We used the receiver operator characteristic (ROC) curve to calculate the cut-off values for viral RNA shedding of potentially infectious influenza A or B viruses using EZR version 1.54 [29]. Viral RNA shedding (log_10_ copies/µL) of all clinical samples collected (first and second visits) were listed with their virus isolation status (yes or no) to estimate the potentially infectious cut-off value. The area under the curve (AUC) of the ROC curve was used to analyze the accuracy of viral RNA shedding to assess potential viral infectivity. AUC value above 0.80 is assessed as a test with good accuracy. The viral RNA shedding in the second visit samples that exceeded the cut-off value was regarded as potentially infectious. We divided the patients according to age, influenza type, and treatment. We calculated the proportion of patients with a detectable viral RNA shedding over the patients who had a second sampling five days after the onset (between 5 to 13 days after onset) in each group. Additionally, the proportion of potential infective virus for patients with and without PA variants was calculated in patients infected with influenza A who received baloxavir. Fisher’s exact test was implemented on two by two or two-by-multiple tables to evaluate proportions, and Bonferroni correction was implemented to adjust *p*-values for two by two pairs as a post hoc test for multiple comparisons.

### Ethics approval

This study was approved by the Ethics Committee of Niigata University (no. #1347, 2018–0317). The present study was conducted following the Declaration of Helsinki (revised in 2013). Written informed consent was obtained from all patients and legal guardians of minors before enrollment.

## Results

### Characteristics of the patients at baseline

**Fig. 1:**
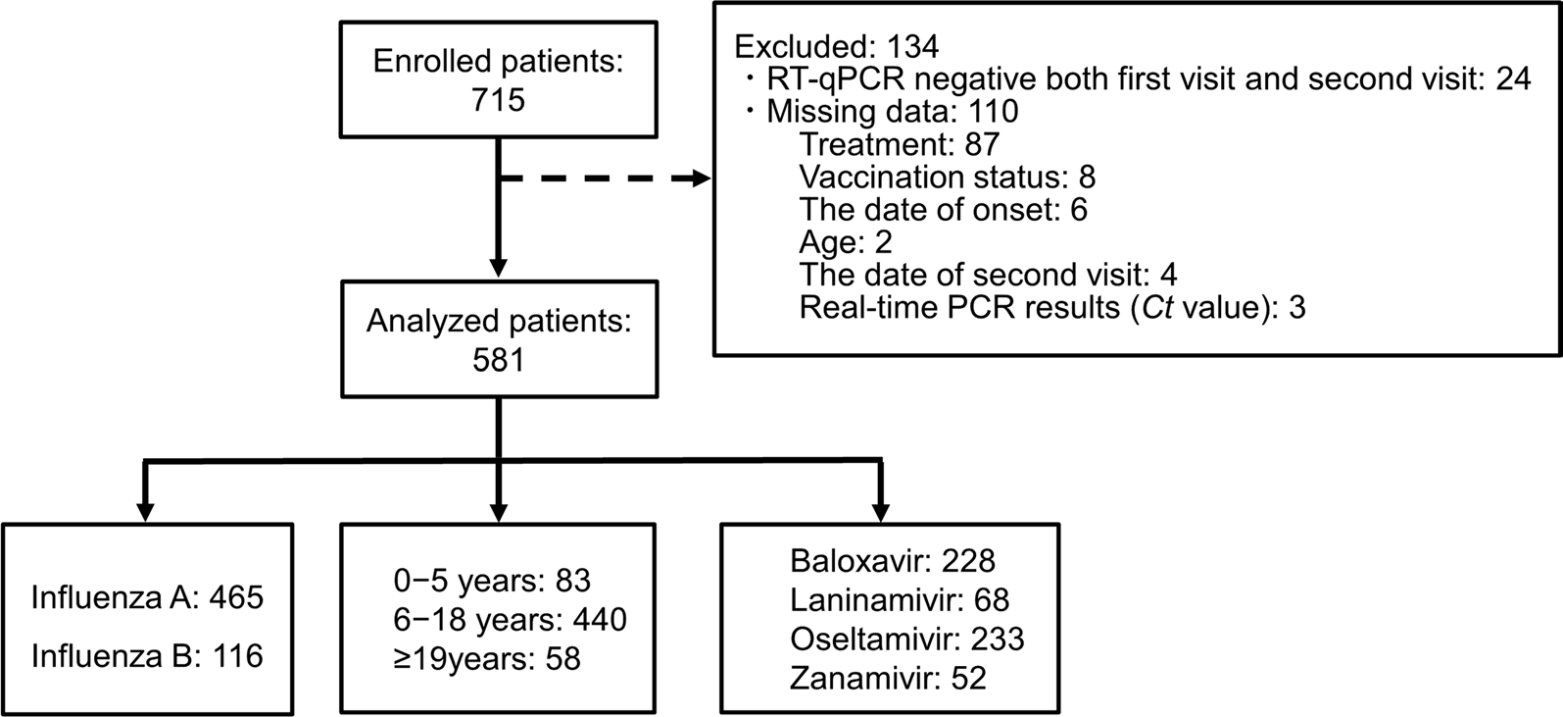
Flowchart

A total of 715 patients at baseline with paired first and second visits were enrolled, and 134 patients were excluded for the reason described in Fig. 1.

Of the 581 patients analyzed, 465 (80.0%) were positive for influenza A (251 A/H1N1pdm09, 199 A/H3N2, and 15 subtypes unspecified) and 116 (20.0%) were influenza B (Table 1). Over half of the patients were male (54.6%, 317/581), and the majority were 6–18 years old (75.7%, 440/581). Among the treatment groups, baloxavir (39.2%, 228/581), and oseltamivir (40.1%, 233/581) were prescribed to more patients than laninamivir (11.7%, 68/581) and zanamivir (9.0%, 52/581). In the vaccination group, over half were unvaccinated patients (54.4%, 316/581). There were no patients found to have NAI-resistant variant viruses (NA/H275Y) after oseltamivir treatment, but 21 (4.5%) patients developed baloxavir-resistant variants viruses (PA/I38T, I38M, I38K, E23K, and E119Q) in the clinical samples of influenza A (9 A/H1N1pdm09 and 12 A/H3N2) after baloxavir treatment [14, 27, 28]. No PA variants were detected in influenza B-infected patients. Patients with PA variants (median 9.67 years [IQR 5.78 years]) were almost the same age as those without PA variants (median 10.33 years [IQR 4.17 years]). The proportion of influenza B infections was higher than influenza A infections among the unvaccinated (69.8% versus 50.5%) patients, but vice versa among the vaccinated (30.2% versus 49.5%; *p* < 0.001) (Table 1). The average viral RNA shedding at the first visit in influenza A infected patients (average ± SD; 4.2 ± 1.3 log_10_ copies/µL) was higher than in influenza B infected patients (3.5 ± 0.9 log_10_ copies/µL) (*p* < 0.001), but that of second visit was similar between patients infected with influenza A (1.0 ± 1.4 log_10_ copies/µL) and B (1.0 ± 1.3 log_10_ copies/µL) (*p* = 0.900). The interval time from onset to first visit in patients was a median of 1.0 days (IQR 0.0–1.0 days) for both influenza A and B but was statistically longer in influenza B (*p* = 0.008) (Table 1). The interval time from the onset to the second visit in patients was similar for influenza A and B, with a median of 5.0 days without statistical significance (IQR 4.0–5.0 days) (*p* = 0.966). The remaining baseline characteristics did not differ among the groups.

**Table 1:** Characteristics of the patients at baseline included in this study

Characteristics	Influenza A	Influenza B	*p*
	*n* = 465	*n* = 116	
Age, average ± SD (years)	12.5 ± 10.8	11.7 ± 10.8	0.509^*a*^
Age group, *n* (%)			0.175^*b*^
0–5 years	69 (14.8%)	14 (12.1%)	
6–18 years	345 (74.2%)	95 (81.9%)	
≥ 19 years	51 (11.0%)	7 (6.0%)	
Gender, *n* (%)			0.104^*b*^
Male	262 (56.3%)	55 (47.4%)	
Female	203 (43.7%)	61 (52.6%)	
Treatment, *n* (%)			0.126^*b*^
Baloxavir	189 (40.6%)	39 (33.6%)	
Laninamivir	58 (12.5%)	10 (8.6%)	
Oseltamivir	181 (38.9%)	52 (44.8%)	
Zanamivir	37 (8.0%)	15 (12.9%)	
Vaccination status, *n* (%)			< 0.001^*b*^
Unvaccinated	235 (50.5%)	81 (69.8%)	
Vaccinated	230 (49.5%)	35 (30.2%)	
Drug resistance substitution, *n* (%)			
NA/H275Y	0 (0.0%)	0 (0.0%)	
PA variants	21 (4.5%)	NA	
Viral RNA shedding, average ± SD (log_10_ copies/µL)			
First visit	4.2 ± 1.3	3.5 ± 0.9	< 0.001^*c*^
Second Visit	1.0 ± 1.4	1.0 ± 1.3	0.860^*a*^
Interval time, Median (IQR) (day)			
From onset to first visit	1 (0–1)	1 (0–1)	0.008^*d*^
From onset to second visit	5 (4–5)	5 (4–5)	0.966^*d*^

SD, standard deviation; NA, not available

^a^Student’s *t* test

^b^Chi-square test

^c^Welch’s *t* test

^d^Mann-Whitney *U* test

### The difference in viral clearance between influenza A and B

The median values of estimated viral clearance between influenza A and B were compared under patient age groups, treatment groups, vaccination status, and the emergence of PA variants (only for influenza A) by the Mann–Whitney *U* test (Table 2).

**Table 2:** Median viral clearance between influenza A and B infected patients

Characteristics	Influenza A	Influenza B	*p*^*a*^
	Median (IQR) (log_10_/day)	Median (IQR) (log_10_/day)	
Age group			
0–5 years	0.56 (0.38–0.85)	0.28 (0.18–0.69)	0.053
6–18 years	0.76 (0.51–1.06)	0.65 (0.38–0.92)	0.029
≥ 19 years	0.87 (0.61–1.01)	0.65 (0.55–0.86)	0.398
Treatment			
Baloxavir	0.81 (0.52–1.12)	0.77 (0.38–1.04)	0.440
Laninamivir	0.76 (0.45–1.06)	0.37 (0.29–0.56)	0.014
Oseltamivir	0.63 (0.44–0.93)	0.64 (0.48–0.86)	0.636
Zanamivir	0.90 (0.65–1.08)	0.55 (0.27–0.77)	0.018
Vaccination status			
Unvaccinated	0.72 (0.49–1.00)	0.63 (0.35–0.89)	0.029
Vaccinated	0.77 (0.49–1.06)	0.65 (0.47–0.87)	0.189
PA variants			
No	0.73 (0.48–1.01)	NA	< 0.001
Yes	0.42 (0.28–0.56)	NA	

IQR, interquartile range; NA, not available

^a^ Mann–Whitney *U* test

In the age groups, the viral clearance of patients infected with influenza A was significantly faster than that of patients infected with influenza B for 0–5 years old (median 0.56 log_10_/day [IQR 0.47 log_10_/day] versus median 0.28 log_10_/day [IQR 0.51 log_10_/day];* p* = 0.053) and 6–18 years old (median 0.76 log_10_/day [IQR 0.55 log_10_/day] versus median 0.65 log_10_/day [IQR 0.54 log_10_/day];* p* = 0.029), respectively, but the clearance in patients ≥ 19 years did not differ between influenza A and B (median 0.87 log_10_/day [IQR 0.40 log_10_/day] versus median 0.65 log_10_/day [IQR 0.31 log_10_/day]; *p* = 0.398).

Patients with influenza A infection who received laninamivir (median 0.76 log_10_/day [IQR 0.61 log_10_/day] versus median 0.37 log_10_/day [IQR 0.27 log_10_/day];* p* = 0.014) or zanamivir (median 0.90 log_10_/day [IQR 0.43 log_10_/day] versus median 0.55 log_10_/day [IQR 0.50 log_10_/day];* p* = 0.018) had faster viral clearance than influenza B infected patients, no difference was found between influenza A and B infected patients who received baloxavir (median 0.81 log_10_/day [IQR 0.60 log_10_/day] versus median 0.77 log_10_/day [IQR 0.66 log_10_/day];* p* = 0.440) and oseltamivir (median 0.63 log_10_/day [IQR 0.49 log_10_/day] versus median 0.64 log_10_/day [IQR 0.38 log_10_/day]; *p* = 0.636).

Unvaccinated patients infected with influenza A demonstrated significantly faster viral clearance than that infected with influenza B (median 0.72 log_10_/day [IQR 0.51 log_10_/day] versus median 0.63 log_10_/day [IQR 0.54 log_10_/day]; *p* = 0.029); however, no difference was observed between the two in the vaccinated group (median 0.77 log_10_/day [IQR 0.57 log_10_/day] versus median 0.65 log_10_/day [IQR 0.40 log_10_/day]; *p* = 0.189).

Patients who did not develop PA variants after baloxavir treatment demonstrated significantly faster viral clearance than patients with emergent PA variants (median 0.73 log_10_/day [IQR 0.53 log_10_/day] versus median 0.42 log_10_/day [IQR 0.28 log_10_/day]; *p* < 0.001) (Table 2). The viral clearance between patients with PA variants and those without were compared within influenza A but not with influenza B. Patients without PA variants analyzed here included all four treatment groups, not only the baloxavir treatment group. In addition, patients with PA variants (median age 9.67 years [IQR 5.78]) were slightly younger than those without PA variants (median age 10.33 years [IQR 4.17]) in influenza A, but no significant difference was observed. Thus, it is unlikely that the age group affected the slower viral clearance of patients who shed PA variants.

### Assessment of influenza viral clearance of patients by age group, treatment, vaccination, and the emergence of PA variants

**Fig. 2:**
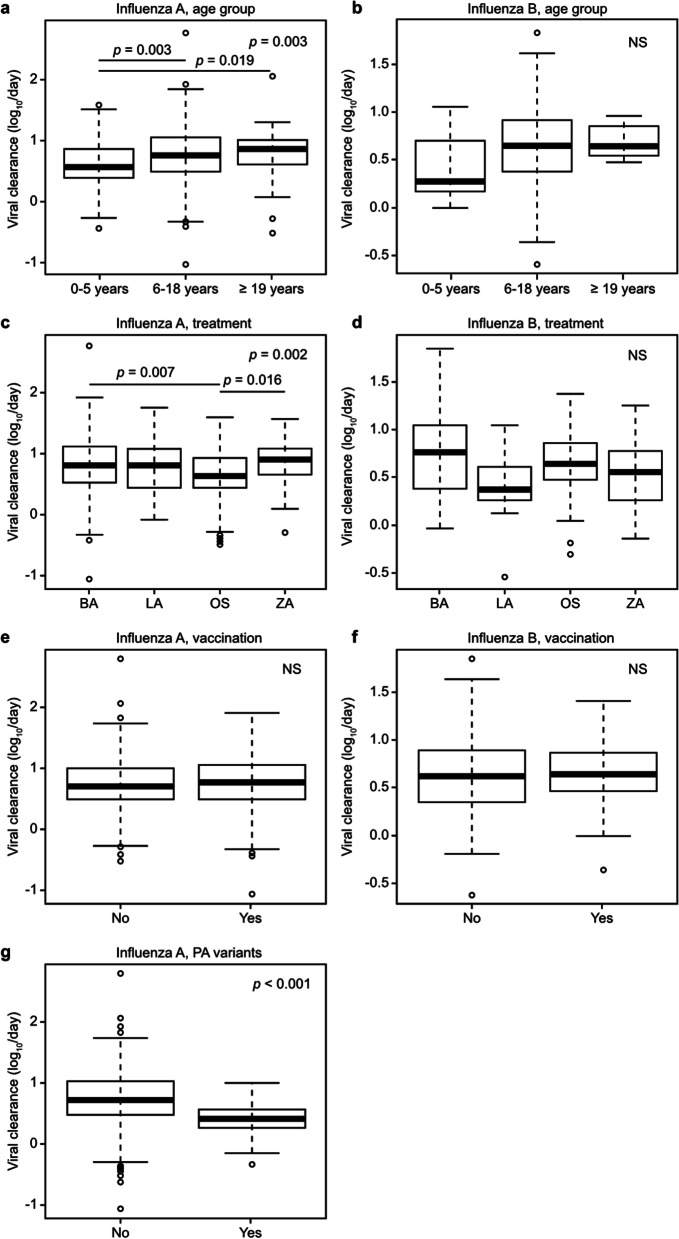
Comparison of influenza viral clearance by age, treatment, vaccination groups with or without PA variants. **a** and **b**; Age groups, **c** and **d**; treatment groups, **e** and **f**; influenza vaccination status, and **g**; emergence of PA variants. Emergence of PA variants was not analyzed for influenza B because of no detection. **a**–**d** were analyzed using Kruskal–Wallis test, and Bonferroni correction was applied as a post-hoc test for each pair; **e**–**g** were analyzed using Mann–Whitney test. The *p*-value or no significance in the upper right corner of each figure was determined using Kruskal–Wallis test or Mann–Whitney test. The *p*-value above the two connected groups in the figure was determined using Bonferroni correction. BA, baloxavir; LA, laninamivir; OS, oseltamivir; ZA, zanamivir; NS, not significant

The association between viral clearance and factors such as age, treatment, vaccination status, or the emergence of PA variants (only for influenza A) was evaluated in patients infected with influenza A or B (Fig. 2).

The median viral clearance of patients 0–5 years was significantly slower than that of patients 6–18 years (*p* = 0.003) and ≥ 19 years old (*p* = 0.019) in influenza A (Fig. 2A and Table 2). No significant difference was found in viral clearance among age groups in influenza B infected patients, maybe due to the smaller number of cases in influenza B (Fig. 2B and Table 1). The median viral clearance of baloxavir and zanamivir-treated patients was significantly faster than that of oseltamivir in influenza A (*p* = 0.007, and *p* = 0.016, respectively) (Fig. 2C). In influenza B, the viral clearance of all four treatment groups did not have a statistical difference (Fig. 2D). Vaccination status did not affect the viral clearance in influenza A-infected patients or influenza B (Fig. 2E, F). Patients with PA variants showed significantly slower viral clearance than those without influenza A (*p* < 0.001) (Fig. 2G).

### Multiple linear regression analysis of influenza viral clearance

Multiple linear regression analysis was performed to determine the association between viral clearance and influenza type, age group, treatment, vaccination status, the emergence of PA variants, and interval time from onset to first visit (Table 3).

**Table 3:** Multiple linear regression analysis of influenza viral clearance

Variables	Partial regression coefficient^*a*^	SE	*t*	*p*	VIF
Type					1.056
Influenza A	Reference	NA	NA	NA	
Influenza B	– 0.134	0.044	– 3.067	0.002	
Age group					1.194
0–5 years	Reference	NA	NA	NA	
6–18 years	0.155	0.052	2.985	0.003	
≥ 19 years	0.170	0.071	2.405	0.016	
Treatment					1.234
Baloxavir	Reference	NA	NA	NA	
Laninamivir	– 0.167	0.058	– 2.883	0.004	
Oseltamivir	– 0.154	0.041	– 3.777	< 0.001	
Zanamivir	– 0.075	0.064	–1.171	0.242	
Vaccination status					1.053
Unvaccinated	Reference	NA	NA	NA	
Vaccinated	0.004	0.035	0.103	0.918	
PA variants					1.072
No	Reference	NA	NA	NA	
Yes	– 0.468	0.094	– 4.965	< 0.001	
Interval time from onset to first visit	0.026	0.026	0.979	0.328	1.009

SE, standard error; VIF, variance inflation factor; NA, not available

^a^ Adjusted *R*^2^ = 0.077; *F* = 6.403

Multivariable analysis showed that viral clearance of patients infected with influenza B was slower by the difference of 0.134 log_10_/day (*p* = 0.002) than that of influenza A. Viral clearance of 6–18 and ≥ 19 years patients were faster by the difference of 0.155 log_10_/day, (*p* = 0.003) and 0.170 log_10_/day (*p* = 0.016), respectively, than that of 0–5 years old. The viral clearance of laninamivir treated patients was 0.167 log_10_/day slower (*p* = 0.004), and oseltamivir-treated patients were 0.154 log_10_/day slower (*p* < 0.001) than that of baloxavir, respectively. In contrast, the viral clearance of zanamivir-treated patients was 0.075 slower without significance (*p* = 0.242). The viral clearance of vaccinated patients did not differ from unvaccinated patients (*p* = 0.918). The viral clearance of patients who shed PA variants was 0.468 log_10_/day slower than that without PA variants (*p* < 0.001). For interval time from onset to the first visit, the viral clearance increased by 0.026 log_10_/day but without significance (*p* = 0.328).

### Residual viral RNA shedding and potential infectivity after 5 days of symptom onset

**Fig. 3:**
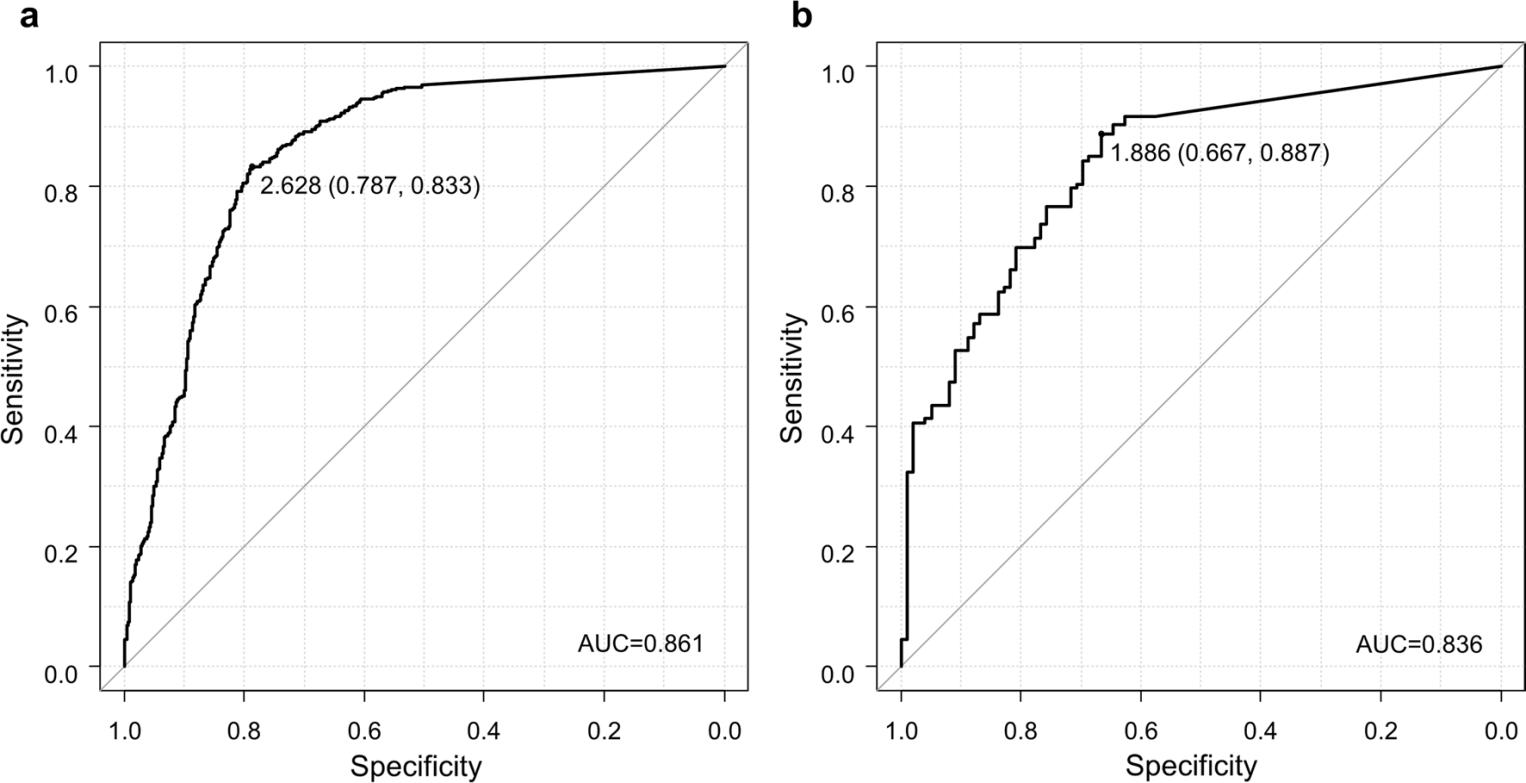
Receiver operating characteristic curves for assessment of CPE using viral RNA shedding. A total of 690 samples from 345 patients with influenza A and 190 samples from 95 patients with influenza B, aged 6–18 years were analyzed. **a**, **b** Represent Influenza A and Influenza B, respectively

To assess the relationship between potential viral infectivity and viral RNA shedding, we first determined the cut-off values for the infectious viral RNA shedding by ROC curve based on the positivity or negativity of virus isolation compared to the viral RNA shedding in each sample. The patient age group of 6–18 years old was focused, and those 0–5 years and > 19 years old were excluded because patients were mainly concentrated in this age group (440/581, 75.7%). As a result, 690 samples (first and second visits) from 345 patients of influenza A and 190 samples (first and second visits) from 95 patients of influenza B collected throughout the study period were analyzed. The calculated cut-off values were 2.628 log_10_ copies/µL for influenza A and 1.886 log_10_ copies/µL for influenza B. The AUC of ROC curves shows that viral RNA shedding accurately assesses potential viral infectivity, 0.861 for influenza A and 0.836 for influenza B, respectively (Fig. 3).

Next, for the analysis of residual RNA shedding and potential infectivity after five days of symptom onset, patients who had a sampling interval between the first and the second with < 5 days or > 14 days were excluded (*n* = 125, 28.4%).

**Fig. 4:**
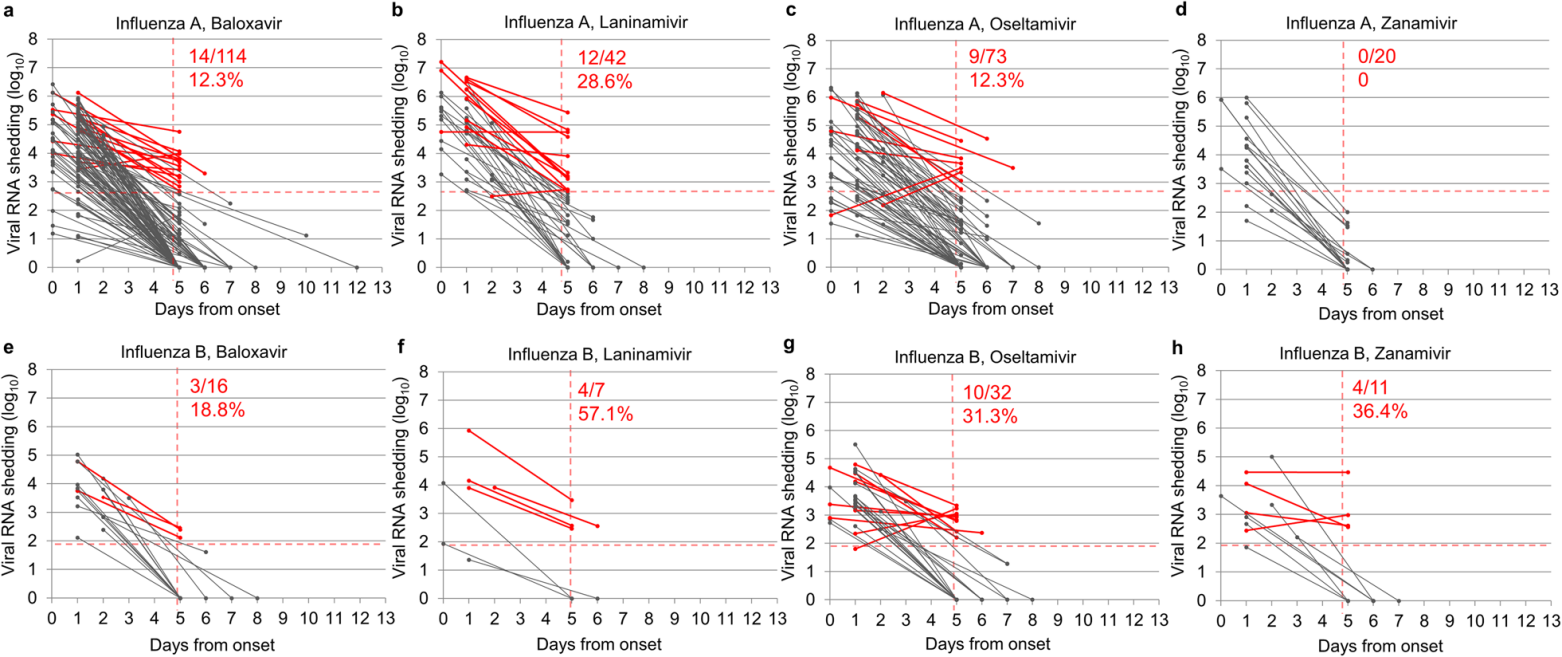
Changes in viral RNA shedding in patients with influenza A and B after treatment. **a** and **e** Patients treated with baloxavir; **b** and **f**, patients treated with laninamivir; **c** and **g**, patients treated with oseltamivir; **d** and **h**, patients treated with zanamivir. The red solid line in each figure represents the paired samples that showed potential infectivity in the second visit collected between 5 to 13th days after symptom onset in the patient. The cut-off value for viral RNA shedding in influenza A and B was 2.628 log_10_ copies/µL and 1.886 log_10_ copies/µL, respectively. The solid gray line represents those with negative potential infectivity below the cut-off. The vertical red dotted line represents the fifth day after symptom onset. The red numeric numbers represent the rate of positive infectivity in the upper part and the corresponding percentage in the lower part

**Fig. 5:**
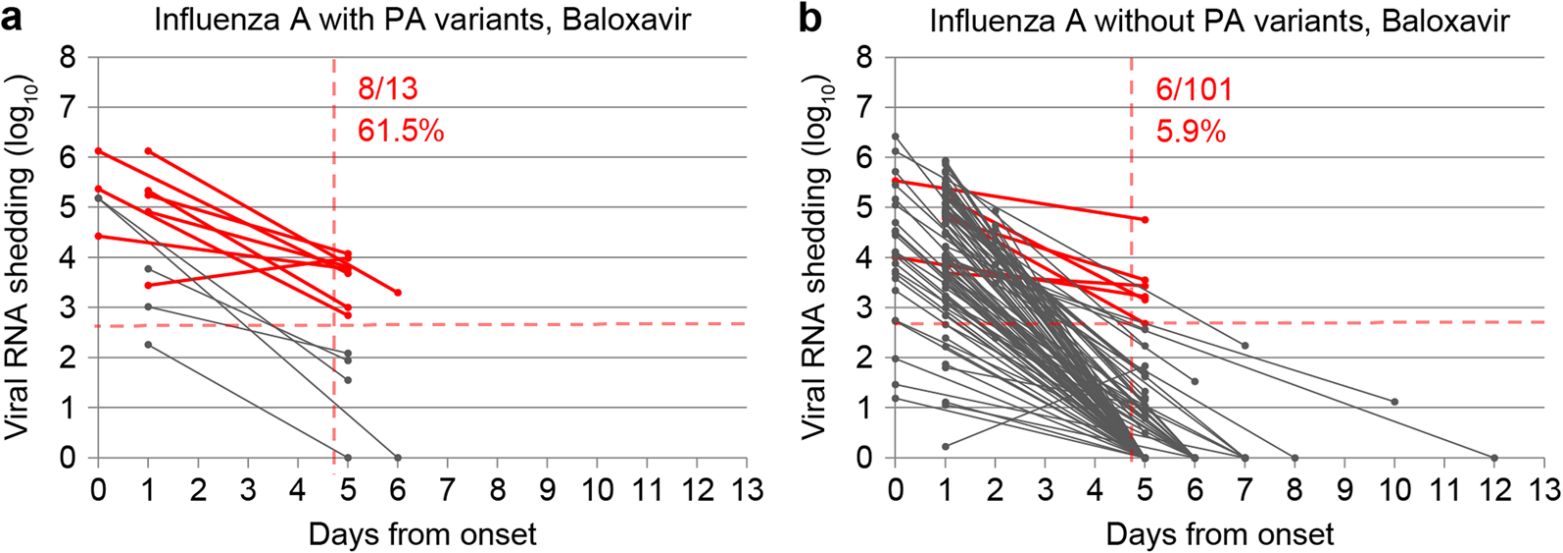
Changes in viral RNA shedding in baloxavir-treated patients with or without PA variants. **a** Patients with PA variants after baloxavir treatment; **b** patients without PA variants after baloxavir treatment. The solid red line in each figure represents the paired samples that showed potential infectivity in the second visit collected between the 5th to 13th days after the patient's symptom onset. The cut-off value for viral RNA shedding in influenza A was 2.628 log_10_ copies/µL. The solid gray line represents those with negative potential infectivity below the cut-off. The vertical red dotted line represents the fifth day after symptom onset. The red numeric numbers represent the rate of positive infectivity in the upper part, and the corresponding percentage in the lower part

A total of 315 patients 6–18 years old were divided by treatment groups (baloxavir, laninamivir, oseltamivir, and zanamivir) and type of influenza (A or B), and the proportion of viral RNA shedding above the potentially infectious cut-off value in the second visit samples was calculated (Fig. 4). In general, patients infected with influenza B (21/66, 31.8%) showed a significantly higher rate of the potential infective virus than influenza A after 5 days of onset (35/249, 14.1%, *p* < 0.001) (Additional file 1: Table S2). Among the four treatment groups, laninamivir treated patients (12/42, 28.6%) showed a higher rate of the potential infective virus than that of zanamivir in influenza A (0/20, 0.0%, *p* = 0.037). Similarly, laninamivir treated patients (4/7, 57.1%) seemed to have a higher rate of the potential infective virus in influenza B compared to other treatment groups; however, statistical difference was not observed because of the small number of patients (Fig. 4 and Additional file 1: Table S2). As was expected from the univariate analysis, influenza A-infected patients who shed emergent PA variants (8/13, 61.5%) after baloxavir treatment showed significantly higher rates of the potential infective virus than those without PA variants (6/101, 5.9%, *p* < 0.001) (Fig. 5).

## Discussion

To the best of our knowledge, this is the first study that collectively compared the viral reduction between a cap-dependent endonuclease inhibitor (baloxavir), and three NAIs (laninamivir, oseltamivir, and zanamivir), against laboratory-confirmed influenza A and B. Multiple linear regression analysis showed that the viral clearance was faster in older (≥ 6 years) patients with influenza A infection and were treated with baloxavir, but it is prolonged with the emergence of PA variants that confer reduced susceptibility to baloxavir (Table 3). In the age group of 6–18 years, approximately 10–30% of patients possessed potential viral infectivity from 5 days, and onward of onset, and the laninamivir treatment groups had a higher rate than the other three treatment groups for influenza A, but these differences were not apparent for influenza B due to the small number of cases (Fig. 4). The patients with PA variants retained higher viral infectivity than those without PA variants in baloxavir-treated influenza A patients (Fig. 5).

The viral clearance of baloxavir was faster than that of NAIs, especially for oseltamivir and laninamivir (Table 3). Similar to our findings, a previous network meta-analysis indicated that baloxavir was more efficacious in controlling the viral RNA shedding than NAIs [30]. However, almost all past studies have compared only baloxavir with oseltamivir but not with other NAIs. A phase 2 randomized control trial in adults showed that by one day after treatment, the decline in infectious virus titers of baloxavir (average 3.36 log_10_ TCID_50_/mL) was significantly higher than that of oseltamivir (average 1.76 log_10_ TCID_50_/mL), and the reduction in viral RNA shedding was also faster in the baloxavir group than in the oseltamivir groups [5]. Similar results were observed in other studies with both adults and children [6, 7]. The faster viral reduction by baloxavir compared to another NAIs, including laninamivir, and not only to oseltamivir, is novel. Laninamivir is a one-time inhalation drug licensed in Japan [2]. Comparative study on viral kinetics for laminamivir with other drugs has not been conducted. Koseki et al. reported that the frequency of clinical biphasic fever for laninamivir was 5.8 times more than zanamivir in children 5–18 years, which may reflect the slower viral clearance in laninamivir treated children [31]. We have demonstrated the similar viral clearance between baloxavir and zanamivir, but the reason remains unclear due to a lack of research.

We found that patient age was positively associated with viral clearance; that is, younger patients had a slower daily virus reduction than older patients. This result may be because children generally have lower pre-existing influenza-specific antibodies than adults [32]. Similar to our findings, a previous study about viral shedding of influenza A, showed that children generally shed a similar amount of virus as adults, but had a longer overall duration and lower rate of decline than adults [33]. Previous studies have shown that the duration of virus shedding is longer in younger patients [8, 34, 35], and that the median viral shedding time of patients < 13 years (median 11 days) is longer than that of patients aged ≥ 13 years (median 7 days) [36]. Additional studies showed that younger children tend to shed greater quantities of influenza virus than older children [37]. These results suggest that younger patients shed more virus and experience a slower daily virus reduction than older patients.

We found that the daily viral reduction (viral clearance) in patients infected with influenza A was faster than influenza B using multiple linear regression analysis (Table 3). We calculated viral clearance using the viral RNA shedding of patients collected at two points during the clinical course, as reported by Rath et al., who showed similar findings regarding the viral clearance of influenza B (median 0.88 log_10_/day), which was slower than oseltamivir sensitive influenza A/H1N1pdm09 (median 1.36 log_10_/day) after oseltamivir treatment [21]. However, our study demonstrated similar viral clearance between patients infected with influenza A and B after oseltamivir treatment in univariate analysis (Table 2). One possible reason was the difference in age composition between the influenza A and B infected patients. In oseltamivir treatment group, the proportion of patients aged 0–5 years (30.9%) in influenza A infection was much higher than that of influenza B (7.7%). Since the daily virus reduction was slower in younger patients, the difference in viral clearance between influenza A and B infected patients in univariate analysis was not significant. Since we have a relatively sufficient number of influenza B (*n* = 52) infected patients compared to influenza A (*n* = 181) for oseltamivir treatment group to draw statistical difference, another possible reason may be related to susceptibility to drugs between influenza A and influenza B. Previous studies have shown that the susceptibility against influenza B compared to A is low in NAIs, 4–15-fold reduction with zanamivir and 3–15-fold with laninamivir, and that with oseltamivir is much larger, 15–45-fold change [38, 39]. On the other hand, the susceptibility of influenza B to baloxavir was just fourfold lower than that of influenza A [40], presumably resulting in smaller difference in viral clearance between influenza A and B after baloxavir treatment.

Herein, we found slower viral clearance in patients infected with PA variants viruses in both uni- and multivariable analyses (Tables 2 and 3, Fig. 3). Additionally, the rate of viral RNA shedding that showed potentially infective virus also supported the slower viral clearance in PA variants than those without (Fig. 5). The PA variants are reported to exhibit reduced susceptibility to baloxavir, and associated with a transient rise in virus titer and prolongation of virus detectability in patients according to the previous findings [12, 41–43]. One of our studies conducted during 2018/2019 season demonstrated the emergence of PA/E23K and I38K/M/S/T variants in 13.5% (13/96) of influenza A infected patients after baloxavir treatment, and the rebound of viral RNA was observed in 13.5% (2/13) patients who shed the PA variants [14]. Thus, our results are compatible with the previous reports of delayed viral clearance when PA variants emerged after baloxavir treatment. In addition to PA variants, resistance variants of NAIs such as NA/H275Y should have a similar effect [10]. However, NA/H275Y variant was not detected in this study maybe because we used virus isolates to screen the NAI-resistant viruses. In some strains of influenza A/H1N1pdm09, the NA/H275Y substituted virus showed reduced viral fitness [44], in contrast to PA/I38T substitution that retained viral fitness relatively well, especially in the case of A/H3N2 [11, 45, 46]. This low viral fitness for NA/H275Y may be one of the reasons why we could not detect it in the second visit samples. Another reason can be the low viral RNA shedding in the second visit samples. As demonstrated by the ROC curve, the threshold of virus isolation was 2.628 log_10_ copies/µL for influenza A, but the average value for the viral RNA shedding at the second visit samples was much lower, 1.0 log_10_ copies/µL (Table 1). Thus, isolating influenza virus in the second visit samples became difficult, and resulted in no detection of NA/H275Y. On the contrary, we detected a total of 21 PA variants in the second visit samples not only due to relatively higher emergence of PA variant after baloxavir treatment, but this screening was directly done from the clinical samples using RT-PCR with a limit of detection of 0.301 log_10_ copies/µL for influenza A, more sensitive than the virus isolation [27].

Approximately 10–30% of children aged 6–18 years had potential infectivity of Additional file 1: Table S2). Additionally, laninamivir-treated patients may shed more infectious viruses than other treatment groups after home stay period in influenza A, although it was not statistically supported in influenza B due to the small number of patients. Our results suggested that the recommended home stay period designated by the School Health and Safety Act may not be sufficient to stop all viral transmission. The School Health and Safety Act in Japan states that children with influenza infection should stay home for at least six days after symptom onset [2, 19]. The purpose of this regulation is to stop transmitting influenza among children and to minimize the size of outbreaks at schools. This rule is applied to elementary schools, junior high schools, high schools, universities, and even workplaces for adults. A certain proportion of patients might remain infectious at the time of returning to school, but the majority of patients became non-infectious (70–90%) (Additional file 1: Table S2), thus appropriate hygiene measurements at school such as wearing masks or frequent ventilation may help stop transmission from infectious patients.

This study has certain limitations. Because there were only two clinical visits, it was impossible to determine the time when the viral RNA shedding was undetectable for the first time. Therefore, we assumed that the viral RNA shedding decreased unidirectionally between the first and second visits, which may cause the daily virus reduction to be slower than the actual situation. Second, the majority of patients had the second sample collection just one day before returning to school; therefore, the actual viral RNA shedding when patients presented to school may have been further reduced. Nevertheless, our study suggests that some patients shed the virus at a lower level at school. Finally, the small number of patients in several groups in the study (e.g., influenza B) compared to others may have affected the main results. Further studies with accumulated case numbers are critically needed to overcome these potential concerns.

Measuring the viral RNA shedding has significant public health implications for controlling transmission via infections. This study verified the recommendations provided by the School Health and Safety Act in Japan and provided evidence for these regulations with regard to the return of children with influenza to school. In general, viral RNA shedding used to be not a major concern for clinicians, unless patients are in severe conditions or immunocompromised. However, after the emergence of coronavirus disease 2019 (COVID-19), it was revealed that the virus shedding of severe acute respiratory syndrome coronavirus 2 (SARS-CoV-2) was longer than the symptomatic period [47, 48], and some patients even caused pre-symptomatic infections [49, 50]. This turned the clinicians to pay more attention to viral RNA shedding in respiratory infections to prevent spread to others. A previous study found that influenza viral RNA shedding is positively correlated with the presence of clinical symptoms [51]. Therefore, one of our future scopes will be to evaluate the association between viral RNA shedding and clinical symptoms. To conclude, our current results can be used as a reference for clinicians in prescribing anti-influenza drugs, and help doctors understand patients who need to focus on to prevent the spread of the virus.

## Supplementary Information

**Additional file 1: Additional Methods.** Quantitative real-time PCR for viral RNA shedding measurement. Genetic analysis to confirm NAIs or baloxavir resistant variants. **Table S1.** Recommended dosage and schedule of antiviral medications for influenza in Japan (as of 2019). **Table S2.** Positivity rate of viral RNA shedding above potentially infectious cut-off after 5 days of onset (6 − 18 years).

## Data Availability

The data that support the findings of this study are available from the corresponding author upon reasonable request.

## Abbreviations

BA: Baloxavir
LA: Laninamivir
OS: Oseltamivir
ZA: Zanamivir
NAIs: Neuraminidase inhibitors
PA: Polymerase acidic
WHO: World Health Organization
RDT: Rapid diagnostic test
cDNA: Complementary DNA
RT-qPCR: Quantitative real-time polymerase chain reaction
MDCK: Madin-Darby canine kidney
CPE: Specific cytopathic effect
SD: Standard deviation
IQR: Interquartile range
SE: Standard error
VIF: Variance inflation factor
NA: Not available
ROC: Receiver operator characteristic
AUC: Area under curve
COVID-19: Coronavirus disease 2019
SARS-CoV-2: Severe acute respiratory syndrome coronavirus 2
