# Supplementary material for: Factors associated with viral RNA shedding and evaluation of potential viral infectivity at returning to school in influenza outpatients after treatment with baloxavir marboxil and neuraminidase inhibitors during 2013/2014–2019/2020 seasons in Japan: an observational study
Source: BMC Infect Dis. 2023 Mar 29;23:188. doi: 10.1186/s12879-023-08140-z (PMC10054210; doi:10.1186/s12879-023-08140-z)
Supplement: Supplementary file 1 — Additional file 1: Additional Methods. Quantitative real-time PCR for viral RNA shedding measurement. Genetic analysis to confirm NAIs or baloxavir resistant variants. Table S1. Recommended dosage and schedule of antiviral medications for influenza in Japan (as of 2019). Table S2. Positivity rate of viral RNA shedding above potentially infectious cut-off after 5 days of onset (6 − 18 years). [file 12879_2023_8140_MOESM1_ESM.docx]

**Additional Files for: Factors associated with viral RNA shedding and evaluation of potential viral infectivity at returning to school in influenza outpatients after treatment with baloxavir marboxil and neuraminidase inhibitors during 2013/14–2019/20 seasons in Japan: an observational study**

**Authors**

Jiaming Li^1^, Keita Wagatsuma^1^, Yuyang Sun^1^, Isamu Sato^2^, Takashi Kawashima^3^, Tadashi Saito^4^, Yasushi Shimada^5^, Yasuhiko Ono^6^, Fujio Kakuya^7^, Nobuo Nagata^8^, Michiyoshi Minato^9^, Naoki Kodo^10^, Eitaro Suzuki^11^, Akito Kitano^12^, Toshihiro Tanaka^13^, Satoshi Aoki^14^, Irina Chon^1^, Wint Wint Phyu^1^, Hisami Watanabe^1^, Reiko Saito^1^

**Author Affiliations**

^1^ Division of International Health (Public Health), Graduate School of Medical and Dental Sciences, Niigata University, Niigata, Japan

^2^ Yoiko Pediatric Clinic, Niigata, Japan

^3^ Kawashima Internal Medicine Clinic, Gunma, Japan

^4^ Tako Central Hospital, Chiba, Japan

^5^ Shimada Children’s Clinic, Kumamoto, Japan

^6^ Ono Pediatric Clinic, Nagasaki, Japan

^7^ Furano Kyokai Hospital, Hokkaido, Japan

^8^ Hiraoka-kouen Pediatric Clinic, Hokkaido, Japan

^9^ Minato Pediatric Clinic, Tokyo, Japan

^10^ Kodo Pediatric Clinic, Kyoto, Japan

^11^ Suzuki Pediatric Clinic, Yamaguchi, Japan

^12^ Kitano Pediatric Clinic, Kumamoto, Japan

^13^ Shizuoka Welfare Hospital, Shizuoka, Japan

^14^ Aoki Pediatric Clinic, Nara, Japan

**Correspondence**

Jiaming Li, Division of International Health (Public Health), Graduate School of Medical and Dental Sciences, Niigata University, Niigata City, Niigata 951-8510, Japan

E-mail address: lijiaming@med.niigata-u.ac.jp

**Additional Methods**

**Quantitative real-time PCR for viral RNA shedding measurement**

Viral RNA was directly extracted from clinical samples using an EXTRAGEN II – DNA/RNA extraction kit (Tosoh Co., Ltd, Tokyo, Japan) and QIAamp Viral RNA Mini Kit (Qiagen, Hilden, Germany) following the manufacturer’s instructions. Viral RNA was transcribed into complementary DNA (cDNA) using the Uni-12 primer (5'- AGCAAAAGCAGG-3') for influenza A and Uni-11primer (5'-AGCAGAAGCRS-3') for influenza B. Quantitative real-time PCR (RT-qPCR) targeting the M gene using TaqMan probes was carried out for the pre- and post-treatment clinical samples to detect viral RNA shedding. The following primers and probes were used to quantify 1) influenza type A: forward primer (5’-TAACCGAGGTCGAAACGTA-3’), reverse primer (5’-GCACGGTGAGCGTGAA-3’), and probe (5’-FAM-TCAGGCCCCCTCAAAGC-Eclipse-3’); 2) influenza type B, forward primer (5'-GCATCTTTTGTTTTTTATCCATTCC-3’), reverse primer (5’-CACAATTGCCTACCTGCTTTCA-3'), and probe (5'-FAM-TGCTAGTTCTGCTTTGCCTTCTCCATCTTCT-Eclipse-3') as previously reported [1].

A standard curve was prepared for quantification using positive controls, serially diluted at eight different concentrations (2.86 copies/µL to 2.86 × 10^7^ copies/µL for influenza A and 2.9 copies/µL to 2.9 × 10^7^ copies/µL for influenza B). Each sample and quantification control were run in duplicates. The viral RNA shedding for each sample was calculated using the standard curve. The threshold for positive cycle value was considered to be over 40. The detection limit for influenza A and influenza B was 2.86 copies/µL and 2.9 copies/µL, respectively. All reactions were performed using an Applied Biosystems 7500 RT-PCR system (Thermo Fisher Scientific KK., Tokyo, Japan).

**Genetic analysis to confirm NAIs or baloxavir-resistant variants**

Conventional PCR was used to amplify cDNA prepared from the clinical samples. The PCR amplicons were purified using QIA quick PCR purification kit (QIAGEN, Hilden, Germany). The sequencing reactions were carried out using a Big Dye Terminator v3.1 cycle sequencing kit (Applied BioSystems, Carlsbad, USA). The sequencing products were run on an ABI Prism 3130xl Genetic Analyzer. Sequence fragments were assembled using Lasergene (DNASTAR, Inc. Madison, WI, USA). The amino acid substitutions in NA and PA proteins were examined and multiple alignments of NA and PA partial sequences were performed using MEGA 6 [2-4].

**Reference**

1. Kondo H, Shobugawa Y, Hibino A, Yagami R, Dapat C, Okazaki M*, et al.* Influenza Virus Shedding in Laninamivir-Treated Children upon Returning to School. Tohoku J Exp Med. 2016;238(2):113–21.

2. Saito R, Osada H, Wagatsuma K, Chon I, Sato I, Kawashima T*, et al.* Duration of fever and symptoms in children after treatment with baloxavir marboxil and oseltamivir during the 2018-2019 season and detection of variant influenza a viruses with polymerase acidic subunit substitutions. Antiviral Res. 2020;183:104951.

3. Wagatsuma K, Saito R, Chon I, Phyu WW, Fujio K, Kawashima T*, et al.* Duration of fever and symptoms in influenza-infected children treated with baloxavir marboxil during the 2019-2020 season in Japan and detection of influenza virus with the PA E23K substitution. Antiviral Res. 2022;201:105310.

4. Osada H, Chon I, Phyu WW, Wagatsuma K, Nagata N, Kawashima T*, et al.* Development of cycling probe based real-time PCR methodology for influenza A viruses possessing the PA/I38T amino acid substitution associated with reduced baloxavir susceptibility. Antiviral Res. 2021;188:105036.

**Supplementary Table 1. Recommended dosage and schedule of antiviral medications for influenza treatment in Japan (as of 2019)**

| Medications | Route of administration | Treatment | |
| --- | --- | --- | --- |
|  |  | Adult | Children |
| Baloxavir | Oral | a. 80 mg single dose (body weight >80 kg)  b. 40 mg single dose (body weight <80 kg) | a. 80 mg single dose (>12 years old body, weight >80 kg)  b. 40 mg single dose (>12 years old, body weight <80 kg)  c. 40 mg single dose (<12 years old, body weight ≥80 kg) *^a^*  d. 20 mg single dose (<12 years old, body weight ≥20 to <40 kg) *^a^*  e. 10 mg single dose (<12 years old, body weight ≥10 to <20 kg) *^a^* |
| Oseltamivir | Oral | 75 mg twice daily, 5 days | a.75 mg twice daily, 5 days (body weight > 37.5 kg) *^b^*  b. 2 mg/kg twice daily, 5 days (maximum 150 mg/day) (body weight < 37.5 kg) *^b^* |
| Zanamivir | Inhalation | 10 mg twice daily, 5 days | 10 mg twice daily, 5 days (> 5 years old) |
| Laninamivir | Inhalation | 40 mg single dose | a. 40 mg single dose (>10 years old)  b. 20 mg single dose (<10 years old) |

*^a^* Administration to patients under 12 years is not recommended.

**Supplementary** **Table 2. Positivity rate of viral RNA shedding above potentially infectious cut-off after 5 days of onset (6−18 years)**

| Type | Positivity rate | Total | Baloxavir | Laninamivir | Oseltamivir | Zanamivir | *p* |
| --- | --- | --- | --- | --- | --- | --- | --- |
| Influenza A | *n* /*N* (%) | 35/249 (14.1%) *^a^* | 14/114 (12.3%) | 12/42 (28.6%) *^b^* | 9/73 (12.3%) | 0/20 (0.0%) *^b^* | 0.014*^c^* |
| Influenza B | *n* /*N* (%) | 21/66 (31.8%) *^a^* | 3/16 (25.0%) | 4/7 (57.1%) | 10/32 (37.5%) | 4/11 (36.4%) | 0.355*^c^* |

*^a^ p* < 0.001 for total influenza A and B patients using Fisher’s exact test.

*^b^* Statistically significant difference was observed between the laninamivir and zanamivir treatment groups of influenza A-infected patients using Bonferroni correction (*p* = 0.037).

*^c^* Fisher's exact test was conducted for the four treatment groups: baloxavir, laninamivir, oseltamivir, and zanamivir.
